# A Comparison between Three Different Techniques Considering Quality Skills, Fatigue and Hand Pain during a Prolonged Infant Resuscitation: A Cross-Over Study with Lifeguards

**DOI:** 10.3390/children9060910

**Published:** 2022-06-17

**Authors:** Roberto Barcala-Furelos, Martín Barcala-Furelos, Francisco Cano-Noguera, Martín Otero-Agra, Alejandra Alonso-Calvete, Santiago Martínez-Isasi, Silvia Aranda-García, Sergio López-García, Antonio Rodríguez-Núñez

**Affiliations:** 1 REMOSS Research Group, Faculty of Education and Sport Sciences, Universidade de Vigo, 36005 Pontevedra, Spain; roberto.barcala.furelos@uvigo.es (R.B.-F.); martinoteroagra@gmail.com (M.O.-A.); alejalonso@uvigo.es (A.A.-C.); 2 Faculty of Health Sciences, Universidad Europea del Atlántico, 39011 Santander, Spain; 3 Faculty of Education, Pontifical University of Salamanca, 37002 Salamanca, Spain; slopezga@upsa.es; 4 INGESPORT Research Group, Department of Physical Activity and Sports, University of Murcia, 30720 San Javier, Spain; francisco.cano@um.es; 5 Facultade de Fisioterapia, Universidade de Vigo, 36005 Pontevedra, Spain; 6 Research, Health and Podiatry Unit, Department of Health Sciences, Faculty of Nursing and Podiatry, Universidade da Coruña, 15071 A Coruña, Spain; smtzisasi@gmail.com; 7 National Institute of Physical Education of Catalonia, Barcelona University, 08038 Barcelona, Spain; silvia.aranda.garcia@gmail.com; 8 Paediatric Intensive Care, Paediatric Intermediate Care and Palliative Care Units, Department of Paediatrics, Hospital Clínico Universitario de Santiago de Compostela, 15706 Santiago de Compostela, Spain; antonio.rodriguez.nunez@sergas.es

**Keywords:** lifeguards, infants, resuscitation, chest compression, two fingers, two thumbs

## Abstract

The aim of the study was to compare the quality of CPR (Q-CPR), as well as the perceived fatigue and hand pain in a prolonged infant cardiopulmonary resuscitation (CPR) performed by lifeguards using three different techniques. A randomized crossover simulation study was used to compare three infant CPR techniques: the two-finger technique (TF); the two-thumb encircling technique (TTE) and the two-thumb-fist technique (TTF). 58 professional lifeguards performed three tests in pairs during a 20-min period of CPR. The rescuers performed compressions and ventilations in 15:2 cycles and changed their roles every 2 min. The variables of analysis were CPR quality components, rate of perceived exertion (RPE) and hand pain with numeric rating scale (NRS). All three techniques showed high Q-CPR results (TF: 86 ± 9%/TTE: 88 ± 9%/TTF: 86 ± 16%), and the TTE showed higher values than the TF (*p* = 0.03). In the RPE analysis, fatigue was not excessive with any of the three techniques (values 20 min between 3.2 for TF, 2.4 in TTE and 2.5 in TTF on a 10-point scale). TF reached a higher value in RPE than TTF in all the intervals analyzed (*p* < 0.05). In relation to NRS, TF showed significantly higher values than TTE and TTF (NRS minute 20 = TF 4.7 vs. TTE 2.5 & TTF 2.2; *p* < 0.001). In conclusion, all techniques have been shown to be effective in high-quality infant CPR in a prolonged resuscitation carried out by lifeguards. However, the two-finger technique is less efficient in relation to fatigue and hand pain compared with two-thumb technique (TF vs. TTF, *p* = 0.01).

## 1. Introduction

Pediatric out-of-hospital cardiac arrest (OHCR) is a rare event [1], but when infant OHCR does occur, drowning is a common cause and is a global public health problem drowning and is a global public health problem [2,3,4] that especially affects toddlers and children aged 0–4 years [5]. Lifeguards usually represent the first line of prevention and intervention in aquatic environments [6,7] and one of their fundamental competencies is cardiopulmonary resuscitation (CPR) [7,8,9,10]. Despite this, only the studies by Weber and Moran focused on pediatric CPR applied by lifeguards [11].

In drowning cardiac arrest, systemic hypoxia is the primary factor [12,13], so conventional CPR including ventilations and compressions is the main recommended strategy [13]. The aim of ventilation is to combat hypoxia [14], and the role of compression is to achieve the necessary cerebral and coronary perfusion [15,16].

For the resuscitation of infants, the pediatric section of the European Resuscitation Guidelines 2021 (ERCG2021) [17] recommends the use of the standard two-finger technique (TF) for one rescuer and the two-thumbs encircling technique (TTE) for two rescuers, although it does open the possibility of other techniques as an alternative to traditional methods when resuscitation conditions or fatigue are limiting factors [17]. Ladny et al. have recently proposed a modification of the TTE technique which they have termed “two-thumb-fist” (TTF) [16], which consists of placing the thumbs together and perpendicular at a 90° angle [18] over the lower third of the infant’s thorax, and applying force with the weight of the body. All these recommendations for pediatric CPR are focused on the most common OHCA situations, either assisted by bystanders or medical teams. However, to our knowledge there is still a lack of evidence related to which techniques can optimize the quality of pediatric CPR with less fatigue and minimizing the injurious consequences for the rescuer, especially in particular resuscitation situations (e.g., drowning and isolated locations). Our study arises from the belief that current infant CPR techniques can be improved [16] and therefore should be studied and analyzed for each context according to the location where the cardiac arrest occurs (e.g., aquatic environments), the type of rescuer (lifeguards) and the resuscitation time (i.e., prolonged).

The objective of this study is to compare three pediatric resuscitation techniques in a lifeguard-assisted out-of-hospital aquatic setting over a prolonged period of time, to determine the quality of resuscitation as well as the perceived fatigue and hand pain of the rescuers.

## 2. Materials and Methods

### 2.1. Study Design

**Figure 1:**
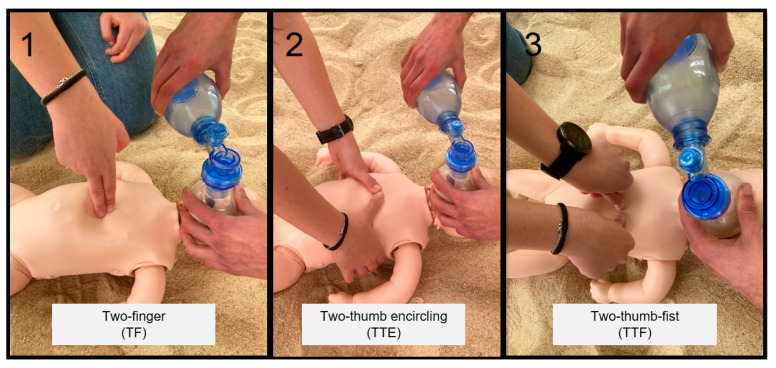
(**1**) Two-finger technique, (**2**) two-thumb encircling, (**3**) two-thumb-fist.

A randomized crossover manikin study was performed to compare the recommended pediatric CPR techniques [17]: two-finger (TF) and two-thumb encircling (TTE) and the new 2-thumb-fist technique (TTF) (Figure 1).

### 2.2. Participants

This study involved a sample of 58 professional lifeguards from 3 Spanish cities (Pontevedra, Santander and Murcia). 40% (*n* = 23) were female and 60% (*n* = 35) were male.

The sample size was based on an assumed minimum of effect size (ES) of 0.25, and error probability of 0.05, and a statistical power of 0.80. These assumptions provided a sample size of 28 study participants computed by G*Power 3.1.9.2 software (Heinrich-Heine-Universität, Düsseldorf, Germany). The final sample was 58 participants, giving a statistical power of 0.99 assuming the effect size and error probability parameters described above. Their mean age was 27 ± 10 years old, their weight was 70 ± 12 kg and their height was 172 ± 9 cm. All participants were informed about the study and gave their written informed consent. The research respected the Helsinki Declaration and the study protocol was approved by the Ethics Committee of the University School of Education and Sport Sciences of the University of Vigo, number 03–0121, date: 18 January 2021.

### 2.3. Study Protocol

**Figure 2:**
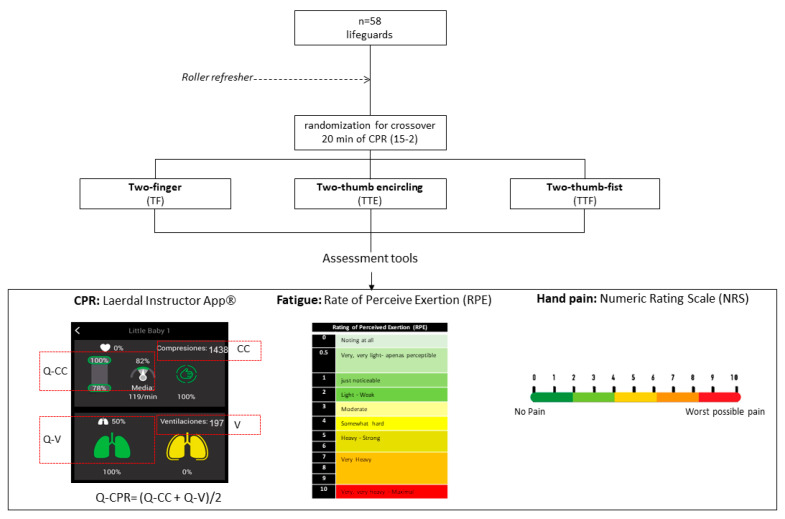
Flow chart design and assessment tools.

The details can be seen in Figure 2.

#### 2.3.1. Step 1 Roller Refresher

Prior to the study, the rescuers received a one-hour training refresher course given by three instructors who are experts in pediatric CPR. All participants were familiarized with the three techniques, with their partner in the resuscitation team and with the manikin on which the tests would be performed.

#### 2.3.2. Step 2 CPR Trial

Each team of rescuers (pair of lifeguards) performed 3 resuscitation tests of 20 min on an infant manikin. The order of the three tests was randomized. To avoid the effect of fatigue, each test was performed 24 h apart. Each team of rescuers (2 lifeguards) followed the sequence as recommended by ERCG2021 [17] for trained responders: After the first 5 rescue breaths, the team followed the sequence of 15 chest compressions (CC) and 2 ventilations (V). One lifeguard performed CC with the randomized technique while the other rescuer delivered V with an infant-size bag-valve-mask (Laerdal. Stavanger, Norway). Every 8 cycles (approximately 2 min) the roles were exchanged between rescuers. The total test time was 20 min.

### 2.4. Variables

#### 2.4.1. Cardiopulmonary Resuscitation Variables

Quality parameters were evaluated and disaggregated into Quality of CC (Q-CC), Quality of V (Q-V) and overall CPR Quality (Q-CPR). Each variable was expressed as a percentage and its calculation is based on the following formulas published in previous studies [19]: Q-CPR = [(Q-CC + Q-V) ÷ 2], Q-CC, calculated using the formula; Q-CC = [%CC with adequate depth + %CC with correct chest recoil + %CC with adequate rate (100–120 CC per minute) ÷ 3] and Q-V = V-C ÷ Number of V × 100. Quantitatively, the number of CC and number of V performed during each test were also recorded.

For data analysis, a Laerdal Little Baby QCPR (Stavanger, Norway) manikin () was used, with the Laerdal Instructor App (Stavanger, Norway) configured according to the ERCG2021 [17]. This model corresponds to a baby of 3 months and approximately 5.5 kg.

#### 2.4.2. Rate of Perceived Exertion (RPE) Parameters

At the perceptual level, the modified rating of perceived effort (RPE) [20] was recorded (measurement of the range 0/10—rest/maximal). Previously, the lifeguards were trained in the knowledge and use of this scale. The RPE was measured individually at five different time points: minute 0, 5, 10, 15 and 20 min into the test.

#### 2.4.3. Hand Pain during CPR

Hand pain was measured using a Numeric rating scale (NRS) whose values range from 0 (no pain) to 10 (worst possible pain) [21,22]. NRS is a scale that is easy to interpret, is intuitive and meets the reliability requirements for pain assessment [22]. The hand pain was also measured individually at five different time points: minute 0, 5, 10, 15 and 20 into the test.

### 2.5. Statistical Analysis

Statistical analysis was performed with IBM SPSS Statistics v.20 for Windows (Armonk, NY, USA). To describe the categorical variable (sex), absolute and relative frequencies were used. To describe the continuous variables of the study, measures of central tendency (mean), dispersion (standard deviation) and confidence estimators (95% confidence intervals) were used. The normality of the distributions was tested using the Kolmogorov-Smirnov and Shapiro-Wilk tests as appropriate and, depending on the results of these analyses, parametric or nonparametric tests were performed. In the parametric analyses, the ANOVA repeated measures test was used and in the nonparametric analyses the Friedman repeated measures test with Bonferroni correction in pairs comparisons was used. For significant comparisons, the effect size (ES) was also calculated with the Rosenthal test. For the interpretation of ES, Cohen’s recommendations were followed: (<0.2: trivial; 0.2–0.5: small; 0.5–0.8: medium; 0.8–1.3: large; >1.3: very large) A significance level of 0.05 was assigned.

## 3. Results

**Figure 3:**
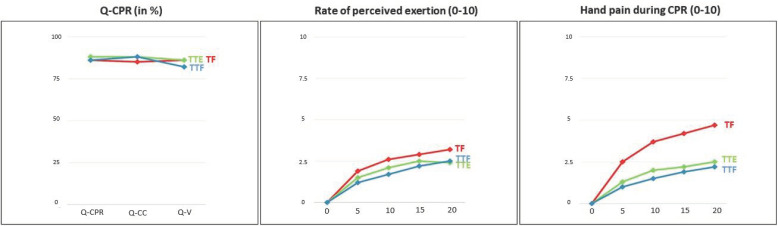
Chart of the results.

The results are based on an analysis of 87 tests, comprising 1740 min of CPR. All the overall results can be seen in Figure 3.

### 3.1. Cardiopulmonary Resuscitation Variables

The results of the CPR tests are shown in Table 1. All techniques obtained high Q-CPR values (TF: 86 ± 9%, TTE: 88 ± 9%/TTF: 86 ± 16%). TTE was statistically superior to TF (TTE vs. TF; *p* = 0.03) although the 2% improvement is not of particular clinical relevance. No differences were found between the techniques in the quality of chest compressions or in the quality of ventilations, with values above 80% in all cases. The quantitative variables of number of CC and number of V were similar, without any statistical significance.

### 3.2. Rate of Perceived Exertion (RPE)

The RPE results are shown in Table 2 and Figure 3. In the intragroup analysis, the significant increase in RPE occurs progressively in all techniques at each control point, compared with baseline state (minute 0). No statistical significance was found in any technique in the intragroup comparison between minutes 15 and 20 (*p* > 0.05). In the comparison of the techniques (intergroup) in each of the sections, statistically significant differences were found between TF and TTF. The TF technique generated a higher RPE from min 5 to min 20 (min 5 *p* = 0.003, min 10 *p* < 0.001, min 15 *p* = 0.02, min 20 *p* = 0.01).

### 3.3. Numeric Rating Scale (NRS) for Hand Pain

The results of hand pain are shown in Table 3 and Figure 3.

In the intragroup analysis, the significant increase in NRS occurred in all three techniques analyzed at each control point (*p* < 0.05); in the comparison of the techniques (intergroup) significantly higher values of hand pain were observed throughout the test when using TF compared to TTE (min 0 vs. 5: *p* = 0.007, min 5 vs. 10: < 0.001, min 10 vs. 15: *p* < 0.001 and min 15 vs. 20: *p* < 0.001) and with TTF (*p* < 0.001 for all intervals). The ES value was medium (0.5 to <0.8) in the comparison of TF with TTE while from min 5 onwards it was large (0.8 to <1.3) in the TF and TTF comparison.

## 4. Discussion

This study was to assess the quality of resuscitation, the perceived fatigue and the hand pain with three pediatric CPR techniques in an aquatic environment assisted by lifeguards. The main findings were: (a) the rescuers are able to maintain a high quality of CPR regardless of the technique employed, (b) perceived fatigue is low in all three techniques, although slightly higher in TF and (c) hand pain using the TF technique is moderate compared to TTE and TTF which was mild.

Survival from pediatric cardiac arrest and a favorable neurological outcome is associated with the duration of CPR [23], as well as witnessed cardiac arrest Delivery of high quality CPR is likely to be another major factor [24]. Therefore, the analysis of the different methods of providing CC in infants has two important challenges; the first one achieving and maintaining high quality CPR without developing fatigue.

Traditionally the TF technique has been recommended when there is only one rescuer [17] and one of the main reasons is to minimize the no-flow time [25], although the time saved compared to TTE is just over half a second [26] and it could be further reduced if two rescuers are carrying out the resuscitation. On the other hand, TTE is recommended when CPR is performed by at least two trained first responders [17], although some studies consider it superior even if performed individually [26,27,28]. One of the main strengths of TTE is the improvement in the depth of CC [26,27,28,29,30,31] compared to TF. This is something that the new TTF method developed by Ladny et al. and Smereka et al. has also achieved [16,18], with the placement of the thumbs at 90°. However there is no superiority in either quality or depth between the methods using the two thumbs TTE or the TTF [32,33]. In our study, we analyzed the Q-CPR in a comprehensive manner. All three techniques achieved values above 85% with no statistically significant differences between groups. The quantification of good CPR in a simulation with manikins has been arbitrarily assumed to be equal to or higher than a value of 70% [34]. One possible explanation is that rescuers have comprehensive training which includes endurance and strength capabilities [35], and their work also specifically requires training in both the lower and upper limbs (including hand muscles), in addition to good physical health to allow CPR to be performed [36] even to allow CPR to be performed under conditions of previous fatigue [8].

One of the analysis points was to find out the intensity of the fatigue and if there were any differences in the technique used. Our findings showed low intensity fatigue (between light and moderate) with no differences between the ways of providing CC. The study by Reynols et al. [37] also found no differences between the techniques analyzed (TT vs. TTE) during 5-min of CPR, but did assume a higher intensity of “hard” fatigue for TT and “somewhat hard” i.e., hard for TTE. Jung et al. did find high fatigue values during a 5-min test with a single-rescuer, especially in the TF technique [38]. Possibly the most relevant difference is the time during which CC is performed without without changing rescuers and it seems that fatigue is also related to the type of victim and increases with greater size [37].

Santos-Folgar et al. analyzed the anatomical area of the upper limb with the greatest fatigue to during 10-min of CPR with the TF technique and found that the only point where fatigue was high was the area between the fingers and the palm of the hand (8 points out of 10) [39]. The Santos-Folgar’s study used the Visual Analogue Scale (VAS) as a fatigue assessment tool. The most common use of this scale is the assessment of pain and the population studied was nursing students, which could perhaps be a confounding bias with fatigue. For this reason, pain was included as a variable using the NRS as a tool because of its better sensitivity [21] in addition to being one of the most widely used scales [40]. As expected, TF resulted in significantly higher pain, which at the end of the test was an increase of 25% compared to the other two techniques. These results are in agreement with previous studies that found the hand to be the least comfortable place during TF [28,38]. The use of the body weight on the fingers in TTF or the pressure of the two hands encircling the infant’s thorax using the anatomical gripper with the thumbs in TTE seems more efficient. Moreover, there is greater involvement of larger muscles or powerful kinetic chains compared to TF whose compression force is lower [29] since it is projected exclusively from the wrist to the two fingers as well as in a smaller contact area [38].

Indeed one of the practical implications of this study is to understand the effects of prolonged pediatric CPR and to optimize it in order to offer the most comfortable alternatives to rescuers, who often operate in remote locations with scarce resources. “Pain cannot be treated if it cannot be assessed” [22] and applied to this study, TTE or TTF would be the preferred choice. The current results should encourage lifeguard organizations to explore these different techniques within their protocols and incorporate fatigue and pain assessment scales to promote best professional practice in the safest, most efficient manner and to avoid injuries that may detract from lifeguard service.

### Limitations of the Study

This study has limitations that should be pointed out. First, it is a simulation study with physically fit rescuers in a controlled context; therefore, in a real situation, with a different first responder profile and in a different resuscitation environment, the results may be different. In this study, not all variables which determine the quality of CPR were collected by an APP software limitation, especially the depth of compression in millimeters, so results must be construed with this important restriction. Another limitation for understand the results was the manikin represents a three-month-old baby. This study should be tested with older and larger toddlers. The limited sample of this study is another important limitation, so further research is needed in order to validate the results obtained. The strength of this study is the novelty and relevance of the topic, as well as the limited evidence in this context. Simulation-based analysis with manikins is useful when the possibility of analysis in situations with real patients has not existed so far.

## 5. Conclusions

All techniques have been shown to be effective in high-quality infant CPR during a prolonged resuscitation provided by lifeguards. However, the two-finger technique is less efficient in relation to rescuers comfort with high fatigue and hand pain compared to two-thumb techniques (TTE and TTF). This study supports the recommendation that the traditional two-finger technique in the context of prolonged resuscitations should not be the preferred option when there is more than one rescuer.

## Figures and Tables

**Table 1:** Results of CPR test (*n* = 29 pairs).

Variables	TF		TTE		TTF		Friedman or ANOVA Test (*p* = 0.05) (In Brackets) Effect Size with Rosenthal Test
	Mean (SD)	CI (95%)	Mean (SD)	CI (95%)	Mean (SD)	CI (95%)	
Q-CPR (%)	86 (9)	[82–89]	88 (9)	[84–91]	86 (16)	[80–92]	TF vs. TTE = 0.03 (ES 0.23)
Q-CC (%)	85 (11)	[81–89]	88 (9)	[84–91]	88 (9)	[85–92]	NS
Q-V (%)	86 (12)	[76–90]	86 (13)	[82–91]	82 (23)	[73–91]	NS
CC	1438 (155)	[1379–1496]	1437 (104)	[1398–1477]	1442 (131)	[1392–1491]	NS
V	197 (21)	[189–205]	197 (14)	[191–202]	197 (17)	[191–204]	NS

TF: two-finger, TTE: two-thumb, TTF: two-thumb-fist Q-CC: Quality of chest compressions; Q-CPR: CPR global quality; Q-V: Quality of ventilations; CC: Number of chest compression; V: Number of ventilations. SD: Standard deviation, CI: 95% Confidence intervals, NS: Not significance.

**Table 2:** Results of Rating of Perceive Exertion (RPE). (*n* = 56; 2 missed).

Variables	TF		TTE		TTF		Friedman Test with Bonferroni Correction (*p* = 0.05)
	Mean (SD)	CI (95%)	Mean (SD)	CI (95%)	Mean (SD)	CI (95%)	
RPE minute 0	0.0 (0.1)	[0.0–0.1]	0.1 (0.3)	[0.0–0.2]	0.0 (0.2)	[0.0–0.1]	NS
RPE minute 5	1.9 (1.6)	[1.5–2.3]	1.5 (1.2)	[1.2–1.8]	1.2 (1.2)	[0.9–1.5]	TF vs. TTF = 0.003 (0.46)
RPE minute 10	2.6 (1.7)	[2.2–3.1]	2.1 (1.3)	[1.8–2.5]	1.7 (1.4)	[1.3–2.1]	TF vs. TTF < 0.001 (0.53)
RPE minute 15	2.9 (1.7)	[2.5–3.4]	2.5 (1.6)	[2.0–2.9]	2.2 (1.6)	[1.8–2.6]	TF vs. TTF = 0.02 (0.37)
RPE minute 20	3.2 (1.8)	[2.7–3.7]	2.4 (1.7)	[2.0–2.9]	2.5 (1.7)	[2.0–2.9]	TF vs. TTF = 0.01 (0.38)
Friedman Test with Bonferroni correction (*p* = 0.05)	0 vs. (5,10,15,20) < 0.001 5 vs. 10 = 0.03 5 vs. (15,20) < 0.001		0 vs. (5,10,15,20) < 0.001 5 vs. (15,20) < 0.001		0 vs. (5,10,15,20) ≤ 0.001 5 vs. (15,20) < 0.001 10 vs. 20 = 0.002		

TF: two-finger, TTE: two-thumb, TTF: two-thumb-fist. RPE: Rating of perceive exertion SD: Standard deviation, CI: 95% Confidence intervals, NS: Not significance.

**Table 3:** Results of Numeric rating scale (NRS). (*n* = 56; 2 missed).

Variables	TF		TTE		TTF		Friedman Test with Bonferroni Correction (*p* = 0.05)
	Mean (SD)	CI (95%)	Mean (SD)	CI (95%)	Mean (SD)	Mean (SD)	
NRS minute 0	0.0 (0.2)	[0.0–0.1]	0.1 (0.1)	[0.0–0.1]	0.0 (0.1)	[0.0–0.1]	NS
NRS minute 5	2.5 (1.8)	[2.0–3.0]	1.3 (1.1)	[1.0–1.6]	1.0 (1.1)	[0.7–1.3]	TF vs. TTE = 0.007 (0.44) TF vs. TTF < 0.001 (0.73)
NRS minute 10	3.7 (2.2)	[3.1–4.2]	2.0 (1.3)	[1.6–2.3]	1.5 (1.4)	[1.1–1.9]	TF vs. TTE < 0.001 (0.63) TF vs. TTF < 0.001 (0.91)
NRS minute 15	4.2 (2.2)	[3.6–4.8]	2.2 (1.3)	[1.8–2.5]	1.9 (1.4)	[1.5–2.2]	TF vs. TTE < 0.001 (0.70) TF vs. TTF < 0.001 (0.90)
NRS minute 20	4.7 (2.5)	[4.0–5.4]	2.5 (1.5)	[2.1–2.9]	2.2 (1.5)	[1.7–2.6]	TF vs. TTE < 0.001 (0.69) TF vs. TTF < 0.001 (0.80)
Friedman Test with Bonferroni correction (*p* = 0.05)	0 vs. (5,10,15,20) ≤ 0.001 5 vs. 10 = 0.013 5 vs. (15,20) < 0.001 10 vs. 20 < 0.001		0 vs. (5,10,15,20) < 0.001 5 vs. 10 = 0.021 5 vs. (15,20) < 0.001 10 vs. 20 = 0.041		0 vs. (5,10,15,20) ≤ 0.001 5 vs. (15,20) < 0.001 10 vs. 20 = 0.003		

TF: two-finger, TTE: two-thumb, TTF: two-thumb-fist, NRS: Numeric rating scale, SD: Standard deviation, CI: 95% Confidence intervals, NS: Not significance.

## Data Availability

Not applicable.
